# Temporal dynamics of cisplatin-induced endothelial senescence and its association with cognitive impairment: insights into the medial prefrontal cortex

**DOI:** 10.3389/fonc.2025.1668372

**Published:** 2025-10-28

**Authors:** Yue Hei, Sheng-nan Kong, Juan-hua Sun, Wei-ping Liu, Qian-fa Long, Hong-mei Zhang

**Affiliations:** ^1^ Department of Oncology, Xijing Hospital of Air Force Military Medical University, Xi’an, China; ^2^ Neurology Hospital, Xi’an People’s Hospital, Northwest University, Xi’an, China; ^3^ Key Laboratory of Central Nervous System Diseases, Xi’an Jiaotong University, Xi’an, China

**Keywords:** endothelial senescence, neuroinflammation, blood-brain barrier, cisplatin-induced cognitive impairment, working memory, prefrontal cortex

## Abstract

**Background:**

Chemotherapy-related cognitive impairment (CRCI) poses significant challenges for cancer survivors, with its underlying mechanisms remaining inadequately understood, particularly in the medial prefrontal cortex (mPFC). This study aimed to investigate the temporal dynamics of cisplatin-induced endothelial senescence, blood-brain barrier (BBB) integrity, neuroinflammation, and their relationship with persistent working memory impairment.

**Methods:**

Adult C57BL/6 mice were treated with cisplatin (2.3 mg/kg) and evaluated at multiple time points post-treatment. Senescence-associated β-galactosidase (SA-β-gal) staining, secretory phenotype (SASP) factors, and expression of p16/p21 proteins were assessed to determine endothelial senescence. BBB integrity was determined using dextran tracer and analysis of tight junction proteins (Claudin-5, ZO-1). Neuroinflammation was investigated via GFAP and Iba1 staining. Cognitive function expression was assessed through the Novel Object Recognition (NOR), Puzzle Box, and modified T-maze tests, focusing on working memory ability.

**Results:**

Cisplatin triggered endothelial senescence in the mPFC, peaking at 1 week, as evidenced by elevated percentage of SA-β-gal positive area, increased SASP factors and p16/p21 expression. BBB dysfunction and glial activation emerged later (peaking at 4 weeks), with significant dextran leakage, reduced Claudin-5/ZO-1 levels and increased GFAP and Iba1 staining respectively. Unlike the early vascular events, showing initial rise followed by slow decline, cisplatin-induced working memory impairment persists through 4–12 weeks, manifested as reduced NOR discrimination index, prolonged times in Puzzle Box, and impaired T-maze performance.

**Conclusion:**

This study delineates a temporal cascade wherein cisplatin induces early mPFC endothelial senescence with delayed BBB dysfunction and neuroinflammation, driving chronic working memory impairment, thus indicating endothelial senescence as early-stage potential target for mitigating CRCI, especially in mPFC-related working memory deficits.

## 1 Introduction

Chemotherapeutic agents are effective in eradicating cancer cells, and they may also inflict toxic effects on the central nervous system, leading to chemotherapy-related cognitive impairment (CRCI), commonly referred to as “chemo-brain” (1, 2). CRCI markedly impacts patients’ quality of life, presenting as declines in memory, executive function, and processing speed (2). Cisplatin is among the most widely used chemotherapeutics across solid tumors and is a well-validated agent for modeling CRCI with robust reproducibility and clinical relevance, and was directly implicated in BBB dysfunction and cognitive deficits in both clinical and preclinical contexts (3). chemotherapy can adversely affect vascular endothelial cells, resulting in decline of tight junction proteins of blood-brain barrier (BBB) (such as Claudin-5 and ZO-1) (4, 5). Besides, these agents can provoke oxidative stress and neuroinflammation (especially activated glial cells), which can further intensify damage to the BBB (6). Consequently, it is imperative to conduct further investigations in to the molecular mechanisms that underpin CRCI.

The prefrontal cortex (PFC) serves as a pivotal brain region responsible for advanced cognitive functions (7). Clinically, patients who received chemotherapy often report challenges in executing complex tasks, and functional magnetic resonance imaging (fMRI) has revealed altered activation patterns within the PFC (8). Models of CRCI also suggest that chemotherapeutic agents can harm neurons and synapses of PFC (9, 10). Given the critical role of the medial prefrontal cortex (mPFC) in cognitive function and its vulnerability of neurotoxic effects, investigating the mechanisms of CRCI in this area is of paramount importance. Current CRCI studies have predominantly focused on overall BBB disruption, neuroinflammation in the hippocampus and white matter regions (4, 11), yet the precise biochemical alterations in the mPFC and associated cognitive performance after cisplatin treatment remains unclear.

Endothelial cell senescence refers to a state where endothelial cells enter an irreversible cell cycle arrest following various stress stimuli (12, 13). This state is characterized by an increased secretion of senescence-associated secretory phenotype (SASP) factors, such as interleukin-6 (IL-6) and tumor necrosis factor-alpha (TNF-α), and an increase in SA-β-gal staining. The senescence of endothelial cells directly impacts the functional integrity of the BBB and may affect the functional state of surrounding cells, thus influencing the neurovascular unit (14). Recent studies (4) further indicate that endothelial cell senescence after chemotherapy may be a significant driving factor of BBB dysfunction in CRCI. This study aims to explore the temporal dynamics of cisplatin-induced endothelial senescence, BBB disruption and neuroinflammation in the mPFC, and delineates a temporal cascade wherein cisplatin induces early mPFC endothelial senescence with delayed BBB dysfunction and neuroinflammation, driving chronic working memory impairment.

## 2 Materials and methods

### 2.1 Animals and study design

**Figure 1:**
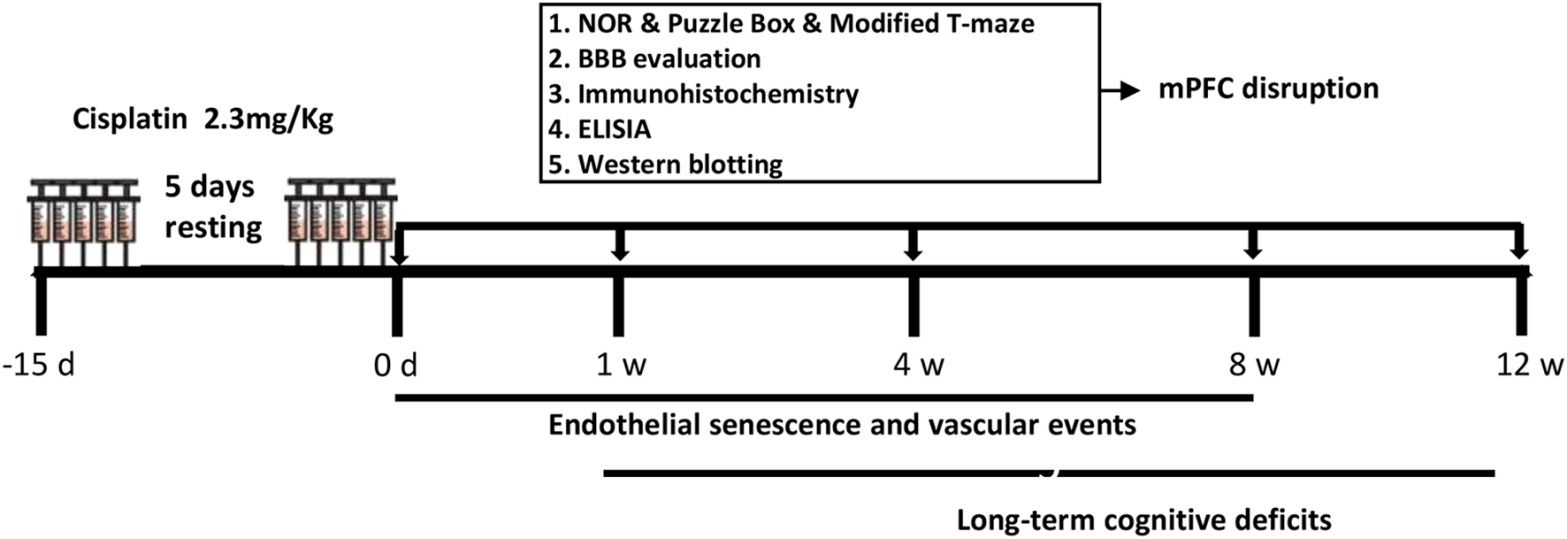
Experimental design. The schematic representation for investigating the temporal changes in endothelial senescence-blood-brain barrier (BBB) dysfunction, glial activation and cisplatin-induced memory impairments. For cisplatin-treatment regimen, mice were treated with cisplatin (2.3 mg/kg) starts from -15 day (d) to 0 d, with 5-day resting period. The assessments of endothelial senescence, BBB disruption and cognitive function were performed at different time points, thus demonstrating the temporal relationship of related phenotypes.

Adult male C57BL/6 mice (8–10 weeks old) were acquired from the Animal Center of the Air Force Military Medical University (Xi’an, China) and maintained under standard laboratory conditions adhering to a 12-hour light/dark cycle, with ad libitum access to food and water. The study protocol received approval from the Animal Care Ethics Committee of the Air Force Military Medical University and was adhered to the National Institutes of Health guidelines for the care and use of laboratory animals. Mice were randomly assigned to different time points and phenotypes with a sample size of N = 4–8 per group. Mice were treated with cisplatin (2.3 mg/kg) with a 5-day resting period. As illustrated in **Figure 1**, assessments of endothelial senescence, blood-brain barrier (BBB) function, and cognitive function were conducted at various time points post-treatment to elucidate the temporal relationship between these phenotypes. Specifically, endothelial senescence was evaluated at 0 day (0 d as control group), 1 week (w), and 4 w using Western blotting, immunohistochemistry, and ELISA. BBB function and glial activation was assessed at 0 d, 1 w, 4 w, and 8 w via tracer immunofluorescence, water content analysis, and Western blotting analysis of tight junction proteins. Cognitive function was evaluated at 0 d, 4 w, 8 w, and 12 w using the Novel Object Recognition (NOR) test, puzzle box test, and modified T-maze test. At the conclusion of each experimental time point, mice were deeply anesthetized by intraperitoneal injection of pentobarbital sodium (150 mg/kg), as evidenced by loss of the pedal reflex, and then decapitated to harvest tissues.

### 2.2 BBB disruption

To evaluate the integrity of the blood-brain barrier (BBB), adult mice were anesthetized with isoflurane (RWD, China)—induction at 4% and maintenance at 1.5% (v/v) in 100% O_2_ via a nose cone, followed by the injection of 5 μl of 10 kDa dextran-tetramethylrhodamine (4 mg/ml, D3312, Invitrogen) into the left ventricle of the heart utilizing a Hamilton syringe. The trace was allowed to circulate for 30 minutes, after which mice were euthanized and decapitated (as above) and tissues were harvested (N = 4). Post-euthanasia, the brains were fixed in 4% paraformaldehyde, subsequently cryopreserved in 30% sucrose solution, and sectioned to a thickness of 12 μm. The resulting sections were analyzed under a fluorescence microscope (Olympus, Japan) to assess the extent of tracer leakage. Furthermore, the brain water content was evaluated by measuring the wet and dry weights of the brain samples (N = 6). The calculation of brain water content was performed using the following formula: Brain water content (%) = [(WW−DW)/WW] × 100 (15).

### 2.3 Immunohistochemistry

Immunohistochemical analysis, in the subsequent section, was performed to evaluate endothelial senescence (N = 4), label endothelial cells during BBB leakage (N = 4) and to stain the activation of glia cells (N = 6). The brain sections underwent fixation in 4% paraformaldehyde followed by permeabilization using 0.5% Triton X-100, and blocking with 5% donkey serum. To identify senescent endothelial cells in the medial prefrontal cortex (mPFC), senescence-associated β-galactosidase (SA-β-gal) staining was executed utilizing a SA-β-gal staining kit (Cell Signaling Technology, #9860, USA) in accordance to the manufacturer’s instructions. Positive area was indicated by blue staining, which included both intense blue and light blue shades that could be differentiated from adjacent area. The percentage of positive area in a 100× microscope field was quantified using ImageJ Software, and comparisons were made across groups. Simultaneously, CD31 immunostaining was conducted to identify endothelial cells. Sections were incubated with primary antibodies against CD31 (BD Pharmingen, 553380, 1:200), GFAP (Immunoway, YM3059, USA) and Iba1 (Immunoway, YM8165, USA) overnight at 4 °C, followed by incubation with secondary antibodies (Alexa Fluor 594/488-conjugated, Invitrogen, 1:200) for 1 hour at room temperature (RT), and were subsequently covered with 4’,6-diamidino-2-phenylindole (DAPI) for analyzing using a confocal microscope (Olympus, Japan). the intensity of the glial staining was expressed as % object area of immuno-reactive cells of the total selected area and the value in the sham group was defined as 100%.

### 2.4 Western blotting

For Western blotting analysis, brain microvessels were isolated (N = 4) from the cerebral cortex and mPFC region (14). This involved dissection followed by digestion with a homogenization buffer containing HEPES, Ca^2+^, collagenase II, and DNase I for 30 minutes, with gentle trituration performed every 10 minutes. Following digestion, the mixture was centrifuged at 1000 g for 5 minutes at 4 °C (16). The resulting cell pellets were resuspended in 20% BSA and centrifuged again at 1000 g for 20 minutes at 4 °C. The final cell pellets were collected for subsequent Western blotting analysis. Protein concentrations were determined using the BCA assay (Bio-Rad Laboratories), and equal quantities of protein (50 μg) were applied to 4-12% Tris-glycine gels, then transferred to nitrocellulose membranes. Membranes were blocked in Tris-buffered saline containing 5% milk for 1 hour at RT. Primary antibodies against p16^INK4a (Abcam, ab16956, 1:1000), p21^Cip1 (Abcam, ab109520, 1:1000), Claudin-5 (Abcam, ab15106, 1:1000), and ZO-1 (Abcam, ab96594, 1:1000) were applied overnight at 4 °C. Peroxidase-conjugated secondary antibodies were incubated for 1 hour at RT. Signals were detected using chemiluminescence (Bio-Rad). The expression levels were quantified by densitometry using ImageJ software.

### 2.5 ELISA for senescence-associated secretory phenotype

Isolation of brain microvessels was carried out as described above. Subsequently, these cells were cultured in endothelial basal medium (EBM)-2, enriched with fetal bovine serum and other growth factors. To quantify the levels of SASP factors, including IL-6, IL-1β, and TNF-α, supernatants from cultured primary brain microvessels were analyzed using enzyme-linked immunosorbent assay (ELISA) kits (R&D Systems, Minneapolis, MN) per the manufacturer’s instructions (N = 6). Absorbance at 450 nm was measured for calculation, with all assays performed in triplicate.

### 2.6 Working memory assessment using NOR, puzzle box, and modified T-maze

Cognitive function was assessed using three behavioral tests: the Novel Object Recognition test (N = 6) (17), the Puzzle Box test (N = 6) (18), and the Modified T-Maze test (N = 6) (7). In the Novel Object Recognition test, mice were exposed to two identical objects (A and B) for 5 minutes during the training phase, followed by a 30-minute rest. During the testing phase, a novel object (B) was placed in the other corner. The test included 5 trials and the time spent exploring each object was recorded using EthoVision XT 10.1 tracking software, and the discrimination index was calculated as (*TB*−*TA*)/(*TB*+*TA*). For the Puzzle Box test, mice were placed in the bright side of a box (61 cm x 30 cm) divided by a barrier with a dark tunnel (4 cm x 2.5 cm) at the bottom. The test included three levels of complexity: an open tunnel (easy), partially blocked by sawdust (intermediate), and completely blocked with tissue (difficult). The time taken for mice to enter the dark compartment was recorded. The test included 11 trials over 4 days, presenting obstacles of increasing difficulty that the mice needed to navigate within 4 minutes per trial. Times exceeding 4 minutes for the first three trials were excluded from data analysis. The modified T-maze was used to assess spatial working memory in mice, prepared from transparent polymethyl methacrylate (PMMA). During each trial, mice were placed at the end of the stem and required to choose one of the two arms (left or right), where they were confined for 30 seconds. The test included 5 trials, recording the chosen arm and reaction time for each trial to calculate the alternation rate. To minimize olfactory cues, the maze was cleaned between trials. Data analysis involved calculating the alternation percentage for each mouse to evaluate their working memory performance, based on the number of entries into the novel (right) versus the familiar (left) arm.

### 2.7 Statistical analysis

Data were analyzed using GraphPad Prism software (version 8.0.0). Results are expressed as mean ± standard error of the mean (SEM). Statistical significance was determined using one-way or two-way analysis of variance (ANOVA) followed by *post-hoc* Bonferroni’s test using SPSS 22.0.0 and for multiple comparisons. For non-parametric data, the Kruskal-Wallis test was employed, followed by the Mann-Whitney U test. A p<0.05 was considered statistically significant. All experiments were performed in triplicate.

## 3 Results

### 3.1 Endothelial senescence in the mPFC region

**Figure 2:**
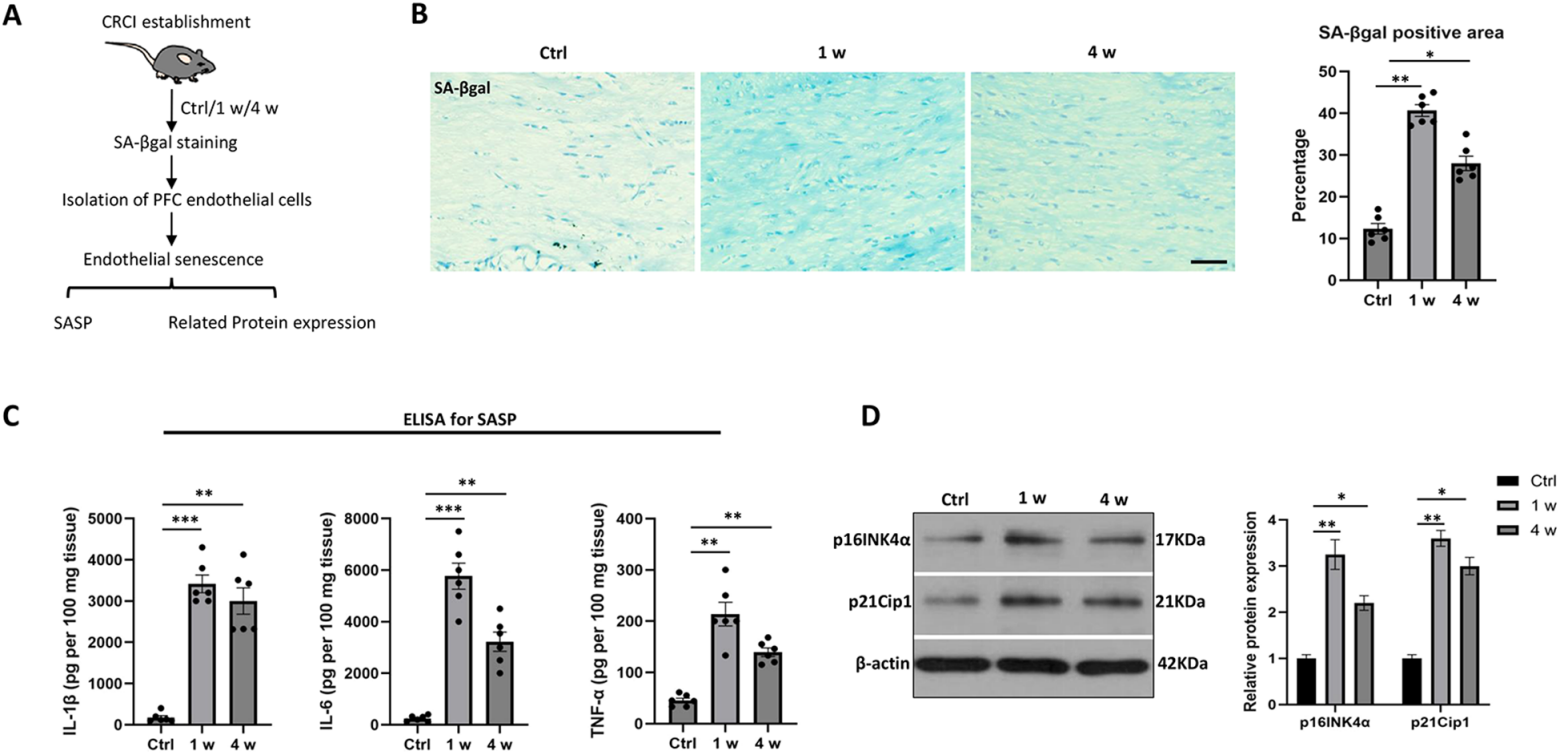
Dynamic changes of endothelial senescence in the mPFC after cisplatin-treatment. **(A)** The experimental design for endothelial senescence assessment. **(B)** Representative images of SA-βgal staining and quantification in the mPFC at 0 day (0 d), 1 week (1 w), and 4 weeks (4 w) post-treatment. The staining indicates the presence of senescent area (blue) The horizontal bar=50 μm. **(C)** ELISA measurements of senescence-associated secretory phenotype (SASP) factors (IL-6, IL-1β, TNF-α) in isolated mPFC endothelial cells at 0 d, 1 w, and 4 w. **(D)** Western blotting analysis of endothelial senescence-related proteins p16INK4α and p21Cip1 in isolated mPFC endothelial cells. β-actin was used as a loading control. Data are presented as mean ± SEM. N = 4 for staining and Western blotting, N = 6 for ELISA test. *p < 0.05, **p < 0.01, ***p < 0.001 compared to control group.

To assess the endothelial senescence induced by cisplatin in the mPFC region, fixed tissue was stained for senescence-associated β-galactosidase (SA-βgal) and isolated endothelial cells from the mPFC were assessed for senescence-associated secretory phenotype (SASP) factors (**Figure 2A**). The results revealed a significant increase in SA-βgal-positive area in mice at 1 week (1 w) and 4 weeks (4 w) after cisplatin administration compared to baseline (0 d), peaking at 1 w (**Figure 2B**). Moreover, ELISA assays demonstrated that the expression levels of SASP factors, including IL-6, IL-1β, and TNF-α, were significantly elevated at 1 w and 4 w (**Figure 2C**). Further analysis through Western blotting of isolated mPFC endothelial cells showed that the expression levels of senescence-related proteins p16^INK4a and p21^Cip1 were significantly upregulated at 1 w and 4 w, peaking at 1 w compared to baseline (control group) (**Figure 2D**). These findings indicate that cisplatin induces early and distinct features of endothelial senescence in the mPFC region.

### 3.2 Delayed BBB permeability changes

**Figure 3:**
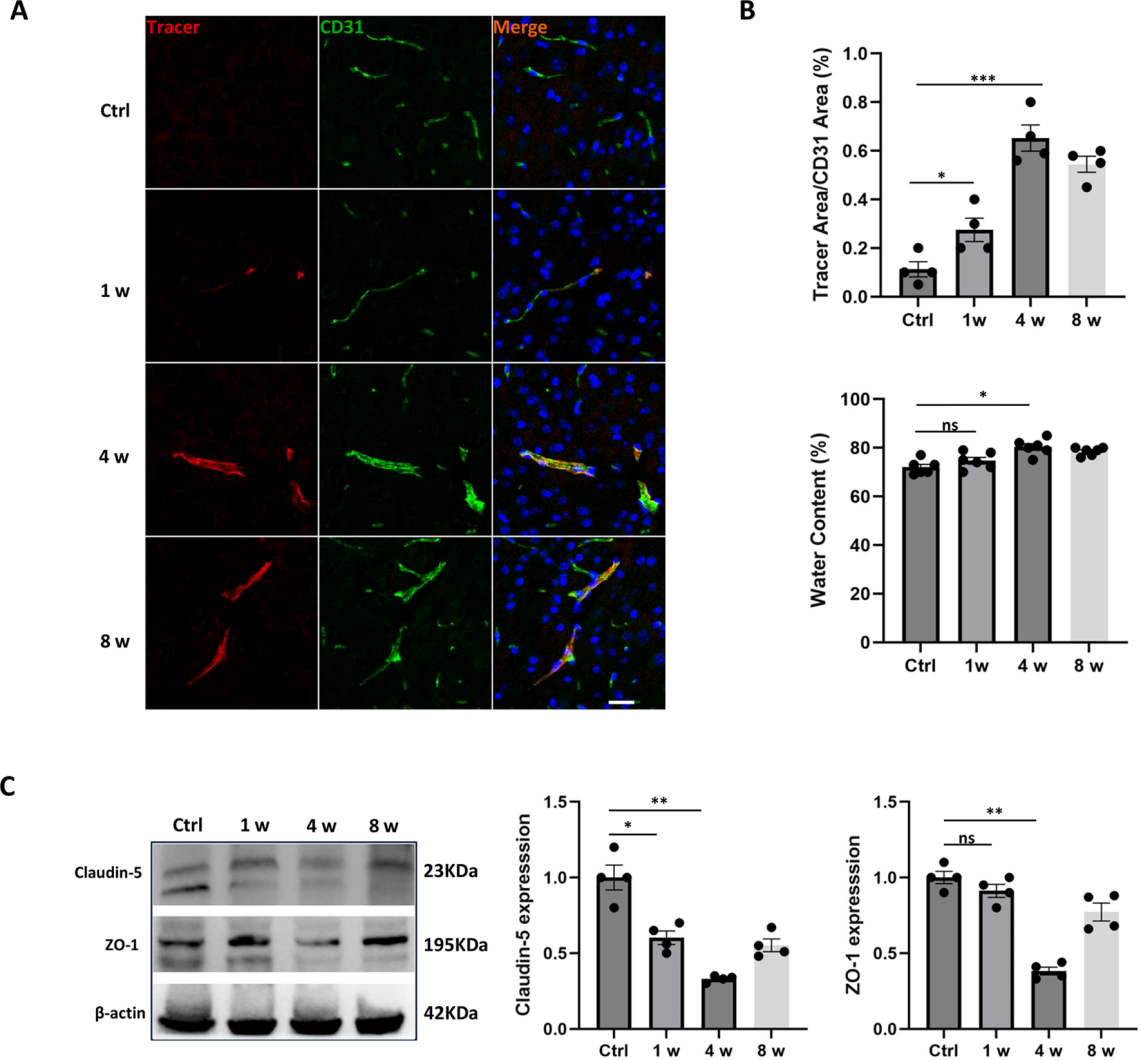
Temporal changes of blood-brain barrier integrity in the mPFC. **(A)** Representative images of immunofluorescence staining for CD31 (green, endothelial marker) and 10 kDa dextran tracer (red) in brain sections at 0 d (control), 1 week (1w), 4 weeks (4w), and 8 weeks (8w) post-treatment. Nuclei are stained with DAPI (blue). The merged image shows the distribution of the tracer within the endothelial areas. The horizontal bar=50 μm. **(B)** The analysis of tracer area/CD31 area to show the BBB leakage, and the water content analysis in brain samples. **(C)** Western blotting analysis of tight junction proteins Claudin-5 and ZO-1 in brain samples. β-actin was used as a loading control. Data are presented as mean ± SEM. N = 4 for immunostaining and Western Blotting, N = 6 for water content. *p < 0.05, **p < 0.01, ***p < 0.001 compared to control group. ns, no significant difference.

To investigate the persistent changes in blood-brain barrier (BBB) permeability induced by cisplatin, we performed immunofluorescence staining using a 10 kDa dextran tracer (red) alongside CD31 (green) to label endothelial cells, assessing BBB leakage. Endothelial senescence occurs prior to the disruption of BBB according to previous findings (4, 13), so we set the observation period for the BBB up to 8 weeks (w). As indicated by the abnormal distribution of the tracer in brain tissue, **Figure 3A** showed significant BBB disruption in mice especially at 4 w and 8 w compared to control group. Furthermore, evaluation of brain water content revealed a significant increase in water content at 4 w (around 80%) following cisplatin treatment, but showing no significant difference at 1 w compared to control group, suggesting that brain edema peaks within 4 w (**Figure 3B**). Western blotting analysis of tight junction proteins Claudin-5 and ZO-1 showed that their expression levels were significantly downregulated at 4 w in comparison to the control group (**Figure 3C**). These results further confirm that BBB dysfunction peaks at four weeks following cisplatin treatment.

### 3.3 Delayed changes of glial activation

**Figure 4:**
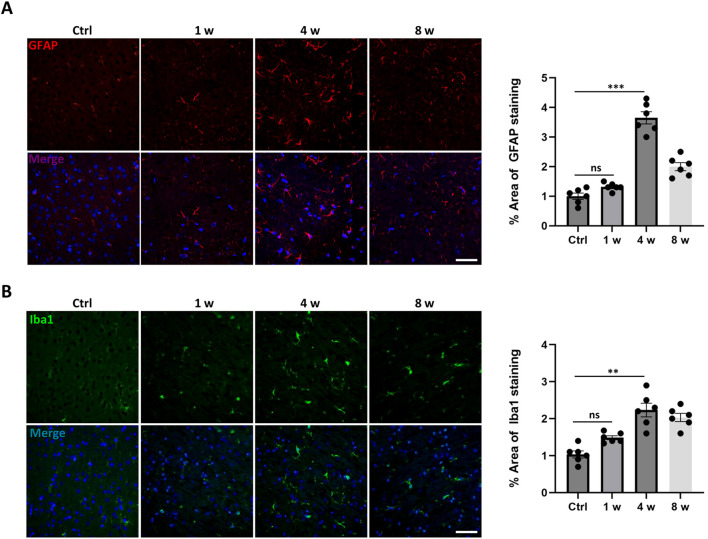
Dynamic changes of neuroinflammation in the mPFC after cisplatin-treatment. **(A)** Representative immunofluorescence images of GFAP-positive astrocytes (red) and merged images with DAPI (blue) at 0 d (control), 1 week (1w), 4 weeks (4w), and 8 weeks (8w) post-cisplatin administration, and the quantitative analysis of GFAP-positive area (% of total area). **(B)** Representative immunofluorescence images of Iba1-positive microglia (green) and merged images with DAPI (blue) at 1w, 4w, and 8w, and the quantitative analysis of Iba1-positive area (% of total area). Scale bar = 50 μm. Data are presented as mean ± SEM. N = 6 for glial staining, **p < 0.01, ***p < 0.001 compared to control group. ns, no significant difference.

To elucidate the temporal dynamics of neuroinflammation in the mPFC region induced by cisplatin, we quantitatively analyzed the activation of astrocytes and microglia using immunofluorescence staining. As shown in **Figure 4**, immunofluorescence staining for GFAP and Iba1 revealed that the activation of astrocytes and microglia respectively exhibited a time-dependent characteristic: in the 4-week group, the area of GFAP-positive signal significantly increased (approximately 3.5%), with cell bodies enlarging and processes extending, indicating a typical activated phenotype (**Figure 4A**). In the 8-week group, the signal intensity decreased but remained elevated (about 2%). Regarding microglia, the Iba1-stained area significantly increased to about 2.5% at 4 weeks, with amoeboid-like morphology (**Figure 4B**). By 8 weeks, there was a decline, yet levels remained above baseline. Notably, the onset and peak events of neuroinflammation occurred after endothelial cell senescence, and the duration of microglial activation seemed to be longer than that of astrocytes.

### 3.4 Persistent working memory impairment

**Figure 5:**
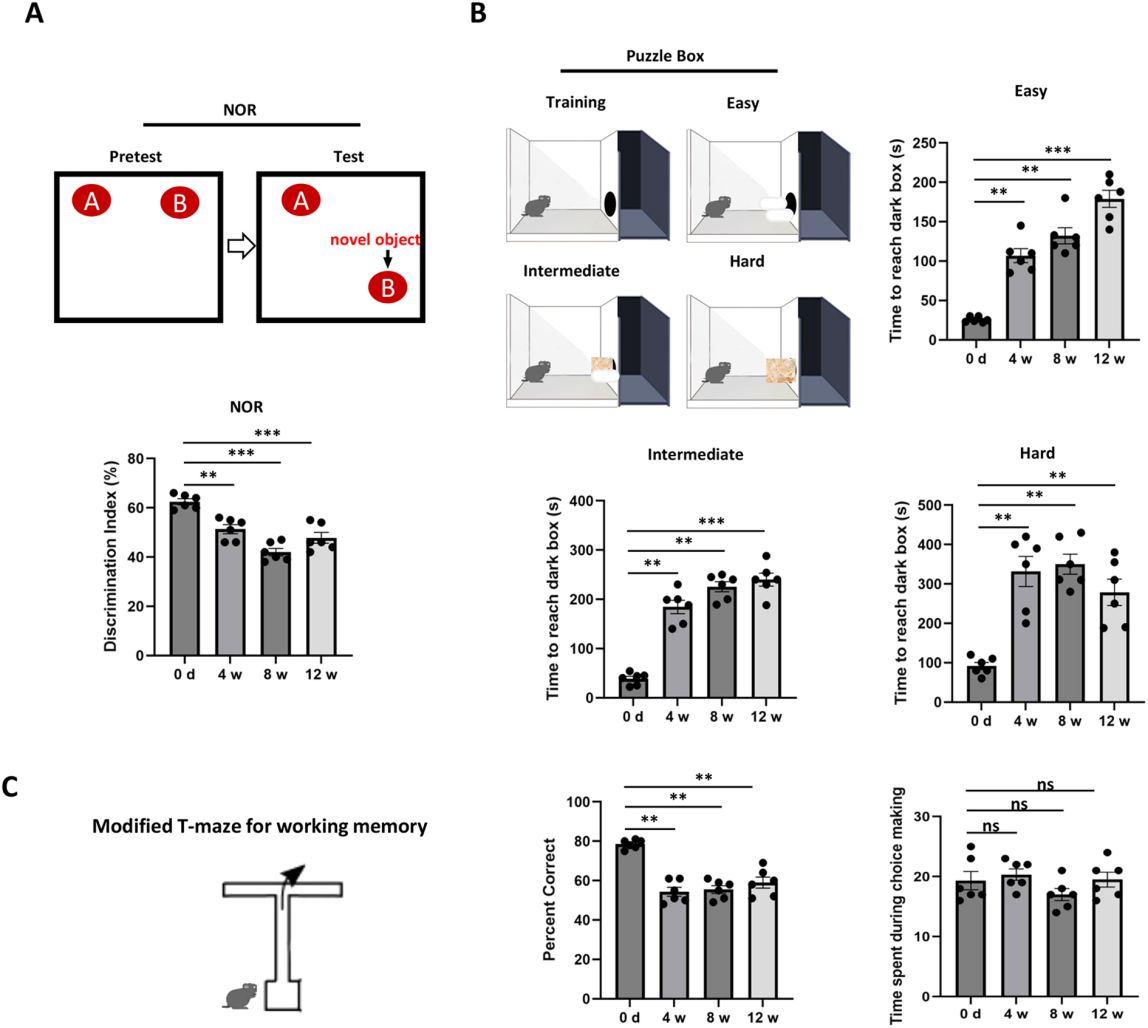
Cognitive function assessment. **(A)** Novel object recognition (NOR) test results at 1 week (1w), 4 weeks (4w), and 8 weeks (8w) post-treatment. The discrimination index was calculated as (TNovel - TFamiliar)/(TNovel + TFamiliar) to assess the preference for the novel object. **(B)** Puzzle box test results at 1w, 4w, and 8w post-treatment. The time taken by mice to enter the dark compartment was recorded. **(C)** Modified T-maze test results at 1w, 4w, and 8w post-treatment. The proportion of entries and time spent in the novel arm (right) and familiar arm (left) were recorded and the maze was cleaned between trials to minimize olfactory cues. N = 6 for all behavioral tests. Repeated trials for NOR and T-maze tests are 5 and for Puzzle box test is 11. Data are presented as mean ± SEM. **p < 0.01, ***p < 0.001 compared to control. “ns” indicates no significant difference between groups.

To assess the mPFC-related cognitive function, we focused on behavioral tests on working memory according to the previous research (7, 18). The novel object recognition (NOR) test demonstrated that the discrimination index for the novel object was significantly reduced in cisplatin-treated mice at 4 w, 8 w, and 12 w compared to control group (0 d), indicating that impaired spatial working memory occurred within 4 w and extended to 12 w (**Figure 5A**). The Puzzle Box test, which assesses PFC-related working ability, showed that cisplatin-treated mice took significantly longer to enter the dark box across different difficulty levels (easy, intermediate, and hard). Notably, there was an upward trend in time spent in the easy and intermediate groups, though results in the hard group were not as stable (**Figure 5B**). The modified T-maze test evaluating spatial working memory revealed that the model group had a significantly lower percentage of correct entries into the novel arm (right) while showing no significant difference in time spent among groups during choice making (**Figure 5C**). Unlike the vascular and neuroinflammation events, which occurred and peaked at certain time points, cognitive impairment demonstrated a persistent decline.

## 4 Discussion

The widespread use of cisplatin, a platinum-based chemotherapeutic agent, has been associated with significant neurotoxic effects, particularly leading to cognitive impairments in cancer survivors (2). Previous research has primarily focused on cognitive changes at the whole-brain level (or hippocampus) (11), with a lack of temporal evidence regarding vascular aging and cognitive impairment in the mPFC region. This study systematically analyzes the temporal dynamics of endothelial cell senescence in the mPFC after cisplatin-treatment, with key findings including: 1) Cisplatin triggers early endothelial cell senescence in the mPFC; 2) A delayed disruption of the BBB and glial activation occurs subsequently (peaking at 4 weeks); 3) Ultimately leading to persistent working memory impairment (4–12 weeks), revealing the potential mechanisms of working memory damage following cisplatin chemotherapy. Our findings highlight a sequential cascade where early endothelial cell senescence precedes BBB disruption and subsequent cognitive decline, illuminating a potentially targetable pathway for therapeutic intervention in patients with CRCI.

Cisplatin has become a cornerstone for the treatment of solid tumors and is widely used in various malignancies, including non-small cell lung cancer (NSCLC), ovarian cancer, and testicular cancer (19). Compared to other chemotherapeutic agents like docetaxel and doxorubicin, cisplatin effectively penetrates the BBB, significantly affecting brain function and causing cognitive impairment (3). The consistency and reproducibility of cisplatin-related cognitive impairment models are robust, with a dosing regimen that is similar to clinical cancer patients (2.3 mg/kg daily) ensuring clinical relevance.

### 4.1 The role of endothelial cell senescence in BBB disruption

Increasing evidence suggests that endothelial cell senescence is a significant driving factor of BBB dysfunction (5, 13, 14). The disruption of the blood-brain barrier (BBB) often correlates with the occurrence of neuroinflammation, characterized by the activation of microglia and the release of pro-inflammatory cytokines. After cisplatin treatment, endothelial cells exhibit a senescence-associated secretory phenotype (SASP), releasing pro-inflammatory cytokines and matrix metalloproteinases that can disrupt the structure and function of the BBB. This study found that cisplatin could induce a senescent phenotype in mPFC endothelial cells as early as 1 w after administration, characterized by an increased percentage of SA-β-gal positive area and significantly elevated levels of SASP factors. This indicates that endothelial cell senescence is a critical early event in cisplatin-induced neurotoxicity. Similarly, studies have shown that paclitaxel can induce senescence in brain microvascular endothelial cells, leading to BBB dysfunction and cognitive impairment (13). By comparison, our study provides a deeper temporal analysis by longitudinally tracking the senescence of endothelial cells at different time points, and observing delayed BBB disruption and glial activation, peaking at 4 weeks. We reveal that cisplatin’s damaging effects on the BBB and glial cells may be subsequent pathological events: (i) BBB leakage permits serum proteins/cytokines to enter parenchyma, activating astrocytes/microglia; (ii) activated glia release cytokines/MMPs that further weaken BBB; (iii) endothelial SASP may prime glia (feed-forward loop) (11, 20). The early onset of endothelial senescence indicates that vascular mechanisms may precede and drive working memory impairment.

### 4.2 Time-course separation of sustained cognitive impairment and vascular events

Damage to the prefrontal cortex can lead to a decline in performance on complex tasks, which verifies the crucial role of the PFC in executive functions and decision-making behaviors (21). Among these, working memory is a system for short-term storage and processing of information, with the PFC being its key brain region (22). Moreover, previous studies further indicated that behavioral experiments such as the modified T-maze provide rapid assessments of working memory using natural mouse behavior while reducing stress responses, serving as important tools for evaluating working memory (7). Here, we find that cognitive impairment persists through 4 to 12 weeks. By evaluating various behavioral paradigms such as the Novel Object Recognition (NOR) task, Puzzle Box, and modified T-maze, we clearly establish the long-term damaging effects of cisplatin on cognitive impairments, especially for working memory. Compared to previous research, this study enhances our understanding of the mechanisms by which cisplatin induces CRCI in several aspects. First, we focused on the mPFC region and identify it necessary for cisplatin-related working memory damage. Second, we revealed a significant time-course separation between cognitive impairment and upstream vascular events, indicating that cognitive impairment occurs after vascular events and glial activation. Unlike the two events, which show an initial rise followed by a slow decline, cognitive impairment displays a sustained decline, suggesting the presence of a chronic neurodegenerative mechanism independent of acute vascular injury. The above results indicates that glial activation can outlast vascular abnormalities and drive chronic inflammation and network dysfunction, accounting for persistent working memory decline even when some vascular markers partially recover. Correspondingly, future investigation may focus on the following aspects: 1) The combined effects of neurotoxicity and vascular endothelial cells; 2) The changes in neurovascular coupling in the context of CRCI. Through these studies, we hope to provide a more comprehensive understanding of the mechanisms underlying CRCI and offer new scientific grounds for developing effective intervention strategies.

### 4.3 Future perspectives: chemo-brain and translational outlook

Targeting endothelial senescence early with senolytics or antioxidants may normalize BBB function by mitigating upstream drivers of senescence-DNA damage, mitochondrial dysfunction coupled with Ca2+ dysregulation, and stress-kinase signaling (p53–p21 and p16 pathways) (17, 23, 24). This framework aligns with redox- and senescence-focused chemotherapy models, and future work should substantiate it using quantitative readouts of oxidative and stress responses (such as DHE/MitoSOX for ROS, 4−HNE/8−OHdG for lipid/DNA oxidation). Furthermore, MSC-based strategies, including iPSC-MSC secretome with proangiogenic and immunosuppressive effects, which could mitigate endothelial injury/inflammation and potentially improve CRCI (25).

In conclusion, the current findings reveal spatiotemporal associations between endothelial senescence, blood-brain barrier integrity, and glial activation. Briefly, Week 1 - endothelial senescence/SASP (mPFC) emerges; Week 4 - BBB leakage/tight-junction loss and peak glial activation; Weeks 4-12 - persistent working memory impairment sustained by glial priming, network and neurovascular-coupling dysfunctions. And this leads to a vicious cycle. Over all, endothelial cell senescence occurs earlier, highlighting it as early-stage potential target for mitigating CRCI, especially in mPFC-related working memory deficits.

## Data Availability

The original contributions presented in the study are included in the article/Supplementary Material. Further inquiries can be directed to the corresponding author.

## References

[1] Mikula-Pietrasik J Witucka A Pakula M Uruski P Begier-Krasinska B Niklas A. Comprehensive review on how platinum- and taxane-based chemotherapy of ovarian cancer affects biology of normal cells. Cell Mol Life Sci. (2019) 76:681–97. doi: 10.1007/s00018-018-2954-1, PMID: 30382284 PMC6514066

[2] Fleming B Edison P Kenny L. Cognitive impairment after cancer treatment: mechanisms, clinical characterization, and management. Bmj. (2023) 380:e071726. doi: 10.1136/bmj-2022-071726, PMID: 36921926

[3] Kim HG Rashid MA Poleschuk M Ullah F Lee SH Kim SH. Cognitive dysfunction in chemobrain: molecular mechanisms and therapeutic implications. BioMed Pharmacother. (2025) 192:118581. doi: 10.1016/j.biopha.2025.118581, PMID: 40997625 PMC13195561

[4] Patai R Csik B Nyul-Toth A Gulej R Vali Kordestan K Chandragiri SS. Persisting blood–brain barrier disruption following cisplatin treatment in a mouse model of chemotherapy-associated cognitive impairment. Geroscience (2025) 47(3):3835–3847. doi: 10.1007/s11357-025-01569-x, PMID: 39982666 PMC12181602

[5] Patai R Kiss T Gulej R Nyul-Toth A Csik B Chandragiri SS. Transcriptomic profiling of senescence effects on blood-brain barrier-related gene expression in brain capillary endothelial cells in a mouse model of paclitaxel-induced chemobrain. Geroscience. (2025) 47(3):3677–3691. doi: 10.1007/s11357-025-01561-5, PMID: 39976844 PMC12181502

[6] Shi DD Huang YH Lai C Dong CM Ho LC Li XY. Ginsenoside rg1 prevents chemotherapy-induced cognitive impairment: associations with microglia-mediated cytokines, neuroinflammation, and neuroplasticity. Mol Neurobiol. (2019) 56:5626–42. doi: 10.1007/s12035-019-1474-9, PMID: 30659419

[7] D’Isa R Comi G Leocani L. Apparatus design and behavioural testing protocol for the evaluation of spatial working memory in mice through the spontaneous alternation t-maze. Sci Rep. (2021) 11:21177. doi: 10.1038/s41598-021-00402-7, PMID: 34707108 PMC8551159

[8] Liu S Ni J Yan F Yin N Li X Ma R. Functional changes of the prefrontal cortex, insula, caudate and associated cognitive impairment (chemobrain) in nsclc patients receiving different chemotherapy regimen. Front Oncol. (2022) 12:1027515. doi: 10.3389/fonc.2022.1027515, PMID: 36408140 PMC9667024

[9] Nguyen LD Fischer TT Ehrlich BE. Pharmacological rescue of cognitive function in a mouse model of chemobrain. Mol Neurodegener. (2021) 16:41. doi: 10.1186/s13024-021-00463-2, PMID: 34174909 PMC8235868

[10] Zhou X Huang Z Zhang J Chen JL Yao PW Mai CL. Chronic oral administration of magnesium-l-threonate prevents oxaliplatin-induced memory and emotional deficits by normalization of tnf-α/nf-κb signaling in rats. Neurosci Bull. (2021) 37:55–69. doi: 10.1007/s12264-020-00563-x, PMID: 32857294 PMC7811972

[11] Yao Z Dong H Zhu J Du L Luo Y Liu Q. Age-related decline in hippocampal tyrosine phosphatase ptpro is a mechanistic factor in chemotherapy-related cognitive impairment. JCI Insight. (2023) 8(14):e166306. doi: 10.1172/jci.insight.166306, PMID: 37485875 PMC10443805

[12] Qi Z Yang W Xue B Chen T Lu X Zhang R. Ros-mediated lysosomal membrane permeabilization and autophagy inhibition regulate bleomycin-induced cellular senescence. Autophagy. (2024) 20:2000–16. doi: 10.1080/15548627.2024.2353548, PMID: 38762757 PMC11346523

[13] Ahire C Nyul Toth A DelFavero J Gulej R Faakye JA Tarantini S. Accelerated cerebromicrovascular senescence contributes to cognitive decline in a mouse model of paclitaxel (taxol)-induced chemobrain. Aging Cell. (2023) 22:e13832. doi: 10.1111/acel.13832, PMID: 37243381 PMC10352561

[14] Lu Y Chen X Liu X Shi Y Wei Z Feng L. Endothelial tfeb signaling-mediated autophagic disturbance initiates microglial activation and cognitive dysfunction. Autophagy. (2023) 19:1803–20. doi: 10.1080/15548627.2022.2162244, PMID: 36588318 PMC10262803

[15] Li W Shi J Yu Z Garcia-Gabilondo M Held A Huang L. Slc22a17 as a cell death–linked regulator of tight junctions in cerebral ischemia. Stroke. (2024) 55:1650–9. doi: 10.1161/STROKEAHA.124.046736, PMID: 38738428

[16] Li G Ma Y Zhang S Lin W Yao X Zhou Y. A mechanistic systems biology model of brain microvascular endothelial cell signaling reveals dynamic pathway-based therapeutic targets for brain ischemia. Redox Biol. (2024) 78:103415. doi: 10.1016/j.redox.2024.103415, PMID: 39520909 PMC11584692

[17] Squillace S Niehoff ML Doyle TM Green M Esposito E Cuzzocrea S. Sphingosine-1-phosphate receptor 1 activation in the central nervous system drives cisplatin-induced cognitive impairment. J Clin Invest. (2022) 132(17):e157738. doi: 10.1172/JCI157738, PMID: 36047496 PMC9433103

[18] Kim T James BT Kahn MC Blanco-Duque C Abdurrob F Islam MR. Gamma entrainment using audiovisual stimuli alleviates chemobrain pathology and cognitive impairment induced by chemotherapy in mice. Sci Transl Med. (2024) 16:eadf4601. doi: 10.1126/scitranslmed.adf4601, PMID: 38446899 PMC11588349

[19] Turrina S Gibelli F De Leo D. Chemotherapy-induced cognitive impairment from the forensic medicine perspective: a review of the updated literature. J Forensic Leg Med. (2020) 76:102070. doi: 10.1016/j.jflm.2020.102070, PMID: 33099125

[20] Ng DQ Hudson C Nguyen T Gupta SK Koh YQ Acharya MM. Dynamin-1 is a potential mediator in cancer-related cognitive impairment. Neurotherapeutics. (2025) 22:e00480. doi: 10.1016/j.neurot.2024.e00480, PMID: 39516074 PMC11742811

[21] Levy R. The prefrontal cortex: from monkey to man. Brain. (2024) 147:794–815. doi: 10.1093/brain/awad389, PMID: 37972282 PMC10907097

[22] Griffin AL. The nucleus reuniens orchestrates prefrontal-hippocampal synchrony during spatial working memory. Neurosci Biobehav Rev. (2021) 128:415–20. doi: 10.1016/j.neubiorev.2021.05.033, PMID: 34217746 PMC8328939

[23] Liu Y Reiken S Dridi H Yuan Q Mohammad KS Trivedi T. Targeting ryanodine receptor type 2 to mitigate chemotherapy-induced neurocognitive impairments in mice. Sci Transl Med. (2023) 15:eadf8977. doi: 10.1126/scitranslmed.adf8977, PMID: 37756377

[24] Alexander JF Seua AV Arroyo LD Ray PR Wangzhou A Heibeta-Luckemann L. Nasal administration of mitochondria reverses chemotherapy-induced cognitive deficits. Theranostics. (2021) 11:3109–30. doi: 10.7150/thno.53474, PMID: 33537077 PMC7847685

[25] Gupta K Perkerson RR Parsons TM Angom R Amerna D Burgess JD. Secretome from ipsc-derived mscs exerts proangiogenic and immunosuppressive effects to alleviate radiation-induced vascular endothelial cell damage. Stem Cell Res Ther. (2024) 15:230. doi: 10.1186/s13287-024-03847-5, PMID: 39075600 PMC11287895

